# Mean Platelet Volume Variability in young patients with Non-ST elevation Acute Myocardial Infarction

**Published:** 2014

**Authors:** AM Andronescu, C Delcea, V Enache, CS Stamate, M Dorobanțu

**Affiliations:** * Internal Medicine and Cardiology Department, “Sf. Pantelimon” Emergency Hospital, Bucharest, Romania; ** Internal Medicine and Cardiology Department, Bucharest Clinical Emergency Hospital, Bucharest, Romania; *** “C. Davila” University of Medicine and Pharmacy, Bucharest, Romania

**Keywords:** mean platelet volume, non-ST elevation myocardial infarction, young, cardiovascular risk factors

## Abstract

**Introduction:** Platelet activation plays an important role in the pathophysiology of non-ST elevation acute coronary syndromes (ACS). Mean platelet volume (MPV), an indicator of platelet reactivity, was previously associated with an increased risk of acute coronary events.

**Objective:** To investigate the MPV variability in young patients presenting with NSTEMI, as compared to young patients with cardiovascular risk factors and no overt ischemic cardiac disease, as well as with elderly patients presenting with NSTEMI.

**Methods:** We analyzed data from 174 patients admitted in our cardiology department between January 2009 and December 2010: 35 patients younger than 45 years of age with NSTEMI, 41 patients younger than 45 without ACS and 98 patients older than 45 with NSTEMI.

**Results:** Young patients with NSTEMI had a significantly higher mean MPV (8.88 ± 1.14fl) than young patients without ACS (8.31 ± 0.37fl, p<0.01), while the older subjects with NSTEMI had the highest mean MPV (9.48 ± 1.35fl, p=0.02). MPV correlated with age (r=0.375, p<0.0001). After a multivariate analysis, elevated levels of MPV were independent predictors of NSTEMI in young patients (odds ratio [OR] 2.75, 95% CI 1.04–7.92, p=0.04), while hypertension (OR 0.34, 95% CI 0.6–1.78, p=0.20), dyslipidemia (OR 1.61, 95% CI 0.17–14.51, p=0.67), obesity (OR 5.77, 95% CI 0.80–41.53, p=0.08) and smoking status (OR 8.97, 95% CI 0.84–95.26, p=0.06) were not.

**Conclusion:** NSTEMI is associated with high MPV in old as well as in young patients. Elevated MPV is predictive for NSTEMI in young patients, separately of a high cardiovascular risk profile.

## Introduction

Platelet function is directly correlated with the mean platelet volume (MPV), a larger volume corresponding to a more active platelet population and a higher thrombotic potential [**1**]. A strong link was found between large, reactive platelets, thrombotic risk [**2**] and the complications of coronary artery disease (CAD) [**3**]. Elevated MPV was associated with an increased risk of acute coronary syndromes (ACS) [**1**,**4**] and acute myocardial infarction (AMI) [**5**], both ST elevation myocardial infarction (STEMI) and non-ST elevation myocardial infarction (NSTEMI)[**6**-**8**]. High MPV was also associated with cardiovascular disease risk and prognosis [**9**-**11**]. Not only CAD, but also uncontrolled cardiovascular risk factors such as hypertension [**12**-**14**], diabetes [**15**,**16**] and metabolic syndrome [**17**] were correlated with an elevated MPV.

An understudied population group, young patients diagnosed with NSTEMI represents a particular subset of ACS subjects, with a distinct risk profile and outcome than older patients [**18**]. MPV was previously described as an independent predictor of AMI in this subgroup [**19**], as young patients presenting with AMI or NSTE-ACS were found to have an increased value compared with healthy subjects [**20**].

## Objective

The aim of our study was to determine the MPV variability in young patients presenting with NSTEMI, as compared to young patients with cardiovascular risk factors and no overt ischemic cardiac disease, as well as in elderly patients presenting with NSTEMI.

## Materials and Methods

Patients and data collection

Data from 174 patients who presented to the Cardiology Department of “Sf. Pantelimon” Emergency Hospital between January 2009 and December 2010 was prospectively analyzed. Our study group consisted of 35 subjects aged 45 or younger diagnosed with NSTEMI by using the 2011 European Society of Cardiology (ESC) Guidelines [**21**], with an aged-matched control group of 41 patients with cardiovascular risk factors but no ACS or documented CAD. Patients included in this control group had no chest pain on admission, no ischemic changes on the ECG, negative troponin and no history of chest pain/stable angina/ACS. A second control group of 98 patients older than 45 years of age, admitted during the same period of time, diagnosed with NSTEMI, was also included. The study complied with the Declaration of Helsinki regarding the investigations in humans and was approved by the Ethic Committee of the hospital; all the patients included gave their informed consent.

Patients with ST elevation myocardial infarction, patients with incomplete risk factor data, as well as patients with infection, anemia, active bleeding, other complete blood count abnormalities (leukocytosis, neutrophilia, lymphopenia) or in whom a full metabolic profile was not available, were excluded. Also patients with antiplatelet therapy in the past month prior to AMI were excluded.

Prior history and newly diagnosed Hypertension, Diabetes and/or Dyslipidemia were used in our analysis. Hypertension was diagnosed according to the ESC guidelines [**22**] as blood pressure above 140/90mmHg or having anti-hypertensive medication. Diabetes mellitus was diagnosed according to the American Diabetes Association 2013 Guidelines as a glycosylated hemoglobin ≥6.5%, at least two values of the fasting plasma glucose ≥126mg/dl, symptoms of hyperglycemia and a random plasma glucose ≥200mg/dl or a 2-h plasma glucose ≥200mg/dl during the oral glucose tolerance test. Dyslipidemia was diagnosed as the lipid profile abnormality that fulfilled the 2011 ESC/EAS guidelines criteria [**23**]. Obesity was defined by a body–mass index (BMI) > 29 kg/m2.

Laboratory analysis

Blood samples for complete blood count (CBC) were acquired from all the patients on admission, before any treatment was administered. The specimens were stored into blood collection tubes with ethylenediaminetetraacetic acid. The CBC was determined within one hour by using a Sysmex XT 1800i Hematology Analyzer. Blood samples collected after at least 8 hours of fasting and in the first 24-48 hours after AMI’s onset were used to determine the lipid profile and fasting glucose levels.

Statistical analysis

SPSS software version 20.0 (SPSS Inc. IL, USA) was used for the statistical analysis. The continuous variables were presented as mean ± standard deviation. Nominal variables were presented as percentages. Mann-Whitney U test was used to compare the MPV in different subgroups. Chi-square and Fisher exact tests were used to describe the association of established parameters and patient outcome (NSTEMI) and to identify the predictors of ACS. Spearman test was used for the correlation of nonparametric variables. The area under the receiver operator characteristic curve (AUROC) and Youden index associated criterion were calculated. A two-tailed p value < 0.05 was considered significant.

## Results

The demographic characteristics of the 35 young patients with NSTEMI were similar to those of 41 young patients without CAD who had the same mean age as well as marked male predominance (**Table 1**). 

**Table 1:** Baseline characteristics of young patients

	**Young patients without AMI **(n=41)	**Young NSTEMI patients **(n=35)	p
Demographics			
Age (years)	39.1 ± 5.3	40.5 ± 3.7	0.22
Male gender (n, %)	35 (85.4%)	32 (91.4%)	0.64
Risk factors			
Hypertension (n, %)	25 (61%)	18 (51.4%)	0.54
Diabetes mellitus (n, %)	0 (0%)	5 (14.3%)	0.01
Dyslipidemia (n, %)	30 (73.2%)	29 (82.9%)	0.46
Obesity (n, %)	8 (19.5%)	16 (45.7%)	0.02
Active smoking (n, %)	21 (51.2%)	27 (77.1%)	0.03
Total risk factors (n, %)			
0	2 (4.8%)	1 (2.9%)	0.87
1	12 (29.3%)	4 (11.4%)	0.10
≥ 2	27 (65.9%)	30 (85.7%)	<0.01

Young patients with NSTEMI had a significant higher prevalence of smoking, diabetes and obesity as compared to the young patients without ACS. 

Platelet number was similar between the three subgroups, but the MPV differed significantly (**Table 2**). 

**Table 2:** Platelet characteristics

	**Young patients without ACS **(n=41)	**Young NSTEMI patients **(n=35)	**Older NSTEMI patients **(n=98)	p1	p2
**Platelets**(n, /mmc)	247000 ± 40977	256342 ± 73639	241816 ± 62055	0.69	0.26
**MPV**(mean ± SD, fl)	8.31 ± 0.37	8.88 ± 1.14	9.48 ± 1.35	**<0.01**	**<0.01**
Correlation of MPV and platelet number	r =-0.408 **p =0.01**	r =-0.151 p =0.52	r =-0.187 p =0.06		
p < 0.05 = significant; p1= p value for comparisons between young patients with and without NSTEMI;
p2= p value for comparisons between young and old NSTEMI patients;

Young patients without ACS had the smallest MPV, while the older subjects with NSTEMI had the highest MPV. MPV correlated with age in the entire patient population (r=0.375, p<0.0001) (**Fig. 1**) and was independent of the platelet number in the NSTEMI patients even though there was a significant correlation in the young non-ACS group between MPV and platelet number (**Table 2**).

**Fig. 1:**
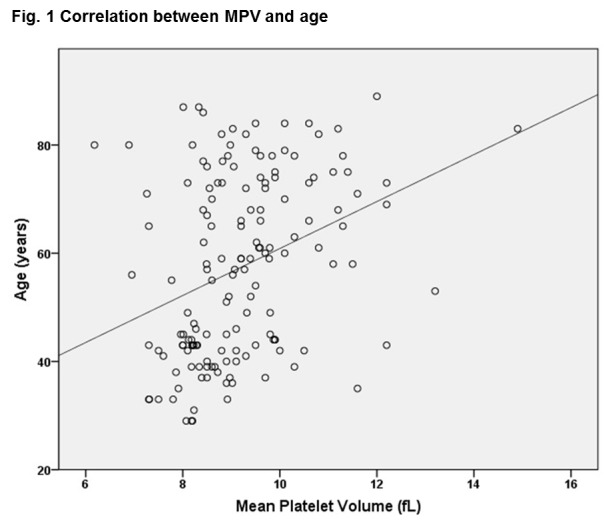
Correlation between MPV and age

To evaluate the relationship between MPV, major cardiovascular risk factors and NSTEMI, the mean MPV of young patients with and without NSTEMI in the subgroups associating hypertension, dyslipidemia, obesity and smoking, were compared. No comparison was possible in the subgroup with diabetes because it only included patients with NSTEMI.

**Fig. 2:**
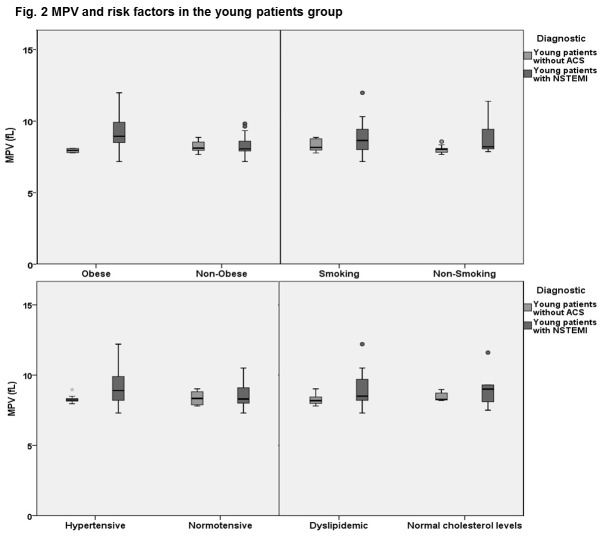
MPV and risk factors in the young patients group

Young hypertensive patients presenting with NSTEMI had a higher mean MPV than those without an ACS, of 9.25 ± 1.25 fl compared to 8.27 ± 0.25 fl (p=0.01). In the young dyslipidemic subgroup, the patients with NSTEMI had a mean MPV of 8.84 ± 1.10 fl, higher than those without an ACS, whose mean MPV was 8.25 ± 0.37 fl (p=0.05). Obese young patients with NSTEMI had a higher MPV than obese young patients without an ACS, of 9.40 ± 1.32 fl compared to 8.08 ± 0.17 fl (p=0.03). Only in the active smoking subgroup of young patients, mean MPV was similar in those with NSTEMI and the control group, of 8.85 ± 1.13 fl vs 8.44 ± 0.41 (p=0.09) (**Fig. 2**). 

Even if in the young NSTEMI group there were significantly more patients with 2 or more risk factors than in those without AMI (**Table 1**), in this high risk subgroup of young patients, mean MPV did not differ significantly between those with and without ACS: 8.98 ±1.18 fl vs. 8.39 ± 0.38 fl (p=0.08).

**Fig. 3:**
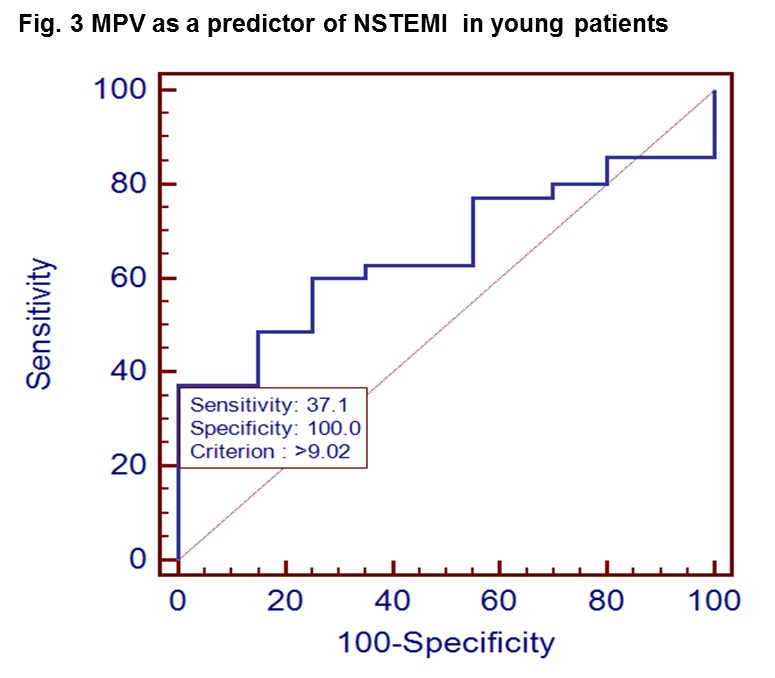
MPV as a predictor of NSTEMI in young patients

ROC curve analysis of the MPV as a marker for NSTEMI retrieved an area under the curve of 0.657 (95% CI 0.517 – 0.780) and a Youden index associated criterion of 9.02 fl (p=0.03) (**Fig. 3**).

Young patients with a MPV above 9.02 fl had a risk ratio of 1.9 (95% CI 1.43 – 2.54) of having NSTEMI (p=0.005). 

After binary logistical regression, an increased MPV was an independent predictor of NSTEMI in our young patient group (odds ratio [OR] 2.75, 95% CI 1.04 – 7.92, p=0.04), while hypertension (OR 0.34, 95% CI 0.6 – 1.78, p=0.20), dyslipidemia (OR 1.61, 95% CI 0.17 – 14.51, p=0.67), obesity (OR 5.77, 95% CI 0.80 – 41.53, p=0.08) and smoking status (OR 8.97, 95% CI 0.84 – 95.26, p=0.06) were not.

Regarding the age discrepancies, there were significant differences between young (less than 45 years) and older NSTEMI patients (**Table 3**). 

**Table 3:** Risk factors prevalence according to age in NSTEMI patients

	**Young NSTEMI patients **(n=35)	**Old NSTEMI patients **(n=98)	p
	Demographics		
Age (years)	40.5 ± 3.7	67.1 ± 12.5	<0.01
Male gender (n, %)	32 (91.4%)	60 (61.2%)	<0.01
	Risk factors		
Hypertension (n, %)	18 (51.4%)	84 (85.7%)	<0.01
Diabetes mellitus (n, %)	5 (14.3%)	37 (37.8%)	0.01
Dyslipidemia (n, %)	29 (82.9%)	68 (69.2%)	0.18
Obesity (n, %)	16 (45.7%)	47 (48%)	0.97
Active smoking (n, %)	27 (77.1%)	27 (27.4%)	<0.01
Total risk factors (n, %)			
0	1 (2.9%)	1 (1%)	0.96
1	4 (11.4%)	19 (19.4%)	0.41
≥ 2	30 (85.7%)	78 (79.6%)	<0.01

**Fig. 4:**
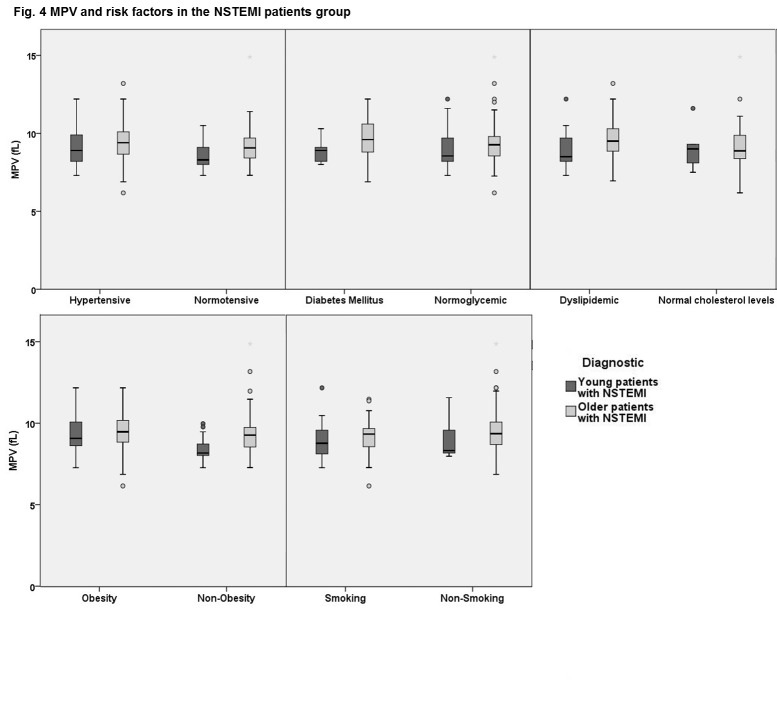
MPV and risk factors in the NSTEMI patients group

As expected, there was a higher male predominance in the younger group. Hypertension and diabetes were significantly more prevalent in the older NSTEMI patients while active smoking and clustering of more than 2 risk factors characterized the young NSTEMI patients. In order to assess the correlation between MPV, age and major risk factors, each subgroup of NSTEMI patients presenting with the five cardiovascular risk factors, was analyzed. A comparable mean MPV was found between the subgroups of young and old NSTEMI patients with hypertension, diabetes or amongst those obese and active smokers. The only significant difference was found in dyslipidemic patients with NSTEMI, where younger subjects had a mean MPV of 8.84 ± 1.10 fl, significantly lower than 9.60 ± 1.22 fl, the mean MPV of the older patients (p=0.005) (**Fig. 4**).

## Discussion

MPV is increased in patients with AMI compared to patients without an ACS [**4**,**8**,**20**] according to studies on populations with an age range between 47 and 68 years old [**3**]. Our aim was to determine if these proportions were valid in a subset of Romanian younger patients (age range between 29 and 45 years old). We found a mean MPV of 8.88 ± 1.14 fL in the young patients with NSTEMI that was significant higher than that found in a control group of young patients with cardiovascular risk factors but no ACS.

In a study with a methodology of MPV measurement similar to ours [**20**], MPV was incriminated as an independent predictor of NSTEMI in young patients, with a RR of 3.1 (95% CI 1.2 – 8.2, p=0.02) after logistical regression analysis, results comparable to those calculated in our young patient population. In another group of young patients with AMI, Ozkan and collaborators also reported higher MPV values in young patients with ACS compared to healthy controls: 8.9 ± 1.0 fL vs. 8.0 ± 0.7 fL [**19**]. In their analysis, the cardiovascular risk profile among young patients with AMI was different than that described in our NSTEMI young patients: 31% had hypertension compared to 51.42% in our study group, 26% had hyperlipidemia compared to 82.86% and 50% were active smokers, compared to 77.14%, respectively. The only risk factor more prevalent among the young patients with ACS in Ozkan’s study was diabetes mellitus, reported in 25% of AMI patients, compared to 14.29% of our NSTEMI subjects.

Interestingly, it seems there could be an age-related correlation between elevated MPV and the major cardiovascular risk factors analyzed in our study: elevated MPV was similar in young smokers with and without NSTEMI while dyslipidemia may have a greater influence on MPV than obesity, diabetes, smoking or hypertension in older NSTEMI patients. Diverging correlations between elevated MPV and cardiovascular risk factors were also reported by prior studies [**9**,**13**-**17**,**24**,**25**].

It was asserted before [**26**] that MPV increases with age; also, that in older AMI patients the high atherosclerotic risk profile may explain the elevated MPV [**3**,**5**,**10**]. Our older NSTEMI patients had the highest MPV; however, the different risk profiles in old versus young NSTEMI patients failed to sustain this correlation in our study.

The main result of our study was that elevated MPV had a high predictive value for NSTEMI in young patients. However, our research could not be generalized to the entire young Romanian NSTEMI population because of some important limitations: the rather small number of patients recruited from only one cardiology center, the lack of diabetic patients in the control group and incomplete data regarding the other confounding factors (coagulation disorders, premature cardiovascular disease history, angiographic findings). Even though we found that a “cut-off” MPV value of 9.02 fl (per our lab reference range) had a statistically significant predictive value for NSTEMI, due to the large variations in the methods of blood sample collection and the type and calibration of the hematologic analyzers [**2**,**10**], we could not extend this value to other centers.

## Conclusion

NSTEMI is associated with an elevated MPV in older as well as in younger patients, separately of a high cardiovascular risk profile. Young patients with NSTEMI have a larger platelet size than the age-matched controls with cardiovascular risk factors and no ACS.

Particularly in the young AMI patients, further extended research was required to identify the relationships between cardiovascular risk factors and this inexpensive, easy to interpret and routinely determined platelet marker.

**Declaration of interest:** No disclosures.
